# The Microenvironment of Decellularized Extracellular Matrix from Heart Failure Myocardium Alters the Balance between Angiogenic and Fibrotic Signals from Stromal Primitive Cells

**DOI:** 10.3390/ijms21217903

**Published:** 2020-10-24

**Authors:** Immacolata Belviso, Francesco Angelini, Franca Di Meglio, Vittorio Picchio, Anna Maria Sacco, Cristina Nocella, Veronica Romano, Daria Nurzynska, Giacomo Frati, Ciro Maiello, Elisa Messina, Stefania Montagnani, Francesca Pagano, Clotilde Castaldo, Isotta Chimenti

**Affiliations:** 1 Department of Public Health, School of Medicine and Surgery, University of Naples Federico II, 80131 Naples, Italy; immacolata.belviso@unina.it (I.B.); franca.dimeglio@unina.it (F.D.M.); annamaria.sacco@unina.it (A.M.S.); veronica.romano@unina.it (V.R.); daria.nurzynska@unina.it (D.N.); stefania.montagnani@unina.it (S.M.); clotilde.castaldo@unina.it (C.C.); 2 Experimental and Clinical Pharmacology Unit, CRO-National Cancer Institute, 33081 Aviano (PN), Italy; francesco.angelini@cro.it; 3 Department of Medical Surgical Sciences and Biotechnologies, Sapienza University, Corso della Repubblica 79, 04100 Latina, Italy; vittorio.picchio@uniroma1.it (V.P.); giacomo.frati@uniroma1.it (G.F.); 4 Department of Clinical, Internal Medicine, Anesthesiology and Cardiovascular Sciences, Sapienza University, 00161 Rome, Italy; cristina.nocella@uniroma1.it; 5 Department of AngioCardioNeurology, IRCCS Neuromed, 86077 Pozzilli, Italy; 6 Department of Cardiovascular Surgery and Transplant, Monaldi Hospital, 80131 Naples, Italy; ciro.maiello@ospedalideicolli.it; 7 Department of Maternal Infantile and Urological Sciences, “Umberto I” Hospital, 00161 Rome, Italy; elisa.messina@uniroma1.it; 8 Institute of Biochemistry and Cell Biology, National Council of Research (IBBC-CNR), 00015 Monterotondo (RM), Italy; francesca.pagano@cnr.it; 9 Mediterranea Cardiocentro, 80122 Napoli, Italy

**Keywords:** extracellular matrix, cardiac stromal cells, microenvironment, cardiac fibrosis, paracrine signals, KDR/VEGFR2

## Abstract

Cardiac adverse remodeling is characterized by biological changes that affect the composition and architecture of the extracellular matrix (ECM). The consequently disrupted signaling can interfere with the balance between cardiogenic and pro-fibrotic phenotype of resident cardiac stromal primitive cells (CPCs). The latter are important players in cardiac homeostasis and can be exploited as therapeutic cells in regenerative medicine. Our aim was to compare the effects of human decellularized native ECM from normal (dECM-NH) or failing hearts (dECM-PH) on human CPCs. CPCs were cultured on dECM sections and characterized for gene expression, immunofluorescence, and paracrine profiles. When cultured on dECM-NH, CPCs significantly upregulated cardiac commitment markers (CX43, NKX2.5), cardioprotective cytokines (bFGF, HGF), and the angiogenesis mediator, NO. When seeded on dECM-PH, instead, CPCs upregulated pro-remodeling cytokines (IGF-2, PDGF-AA, TGF-β) and the oxidative stress molecule H_2_O_2_. Interestingly, culture on dECM-PH was associated with impaired paracrine support to angiogenesis, and increased expression of the vascular endothelial growth factor (VEGF)-sequestering decoy isoform of the KDR/VEGFR2 receptor. Our results suggest that resident CPCs exposed to the pathological microenvironment of remodeling ECM partially lose their paracrine angiogenic properties and release more pro-fibrotic cytokines. These observations shed novel insights on the crosstalk between ECM and stromal CPCs, suggesting also a cautious use of non-healthy decellularized myocardium for cardiac tissue engineering approaches.

## 1. Introduction

Despite remarkable progress in terms of early diagnosis and prevention, heart failure (HF) is still a leading cause of death in Western countries [1], with ischemic heart disease (IHD) being a main etiological cause with its multifaceted pathogenetic mechanisms [2]. IHD affects the viability and function of cardiomyocytes [3] and other resident cells (e.g., smooth muscle, endothelial, and stromal), leading also to significant changes in the composition and architecture of the extracellular matrix (ECM). Consequently, the crosstalk between ECM and resident cells in IHD and HF produces a detrimental microenvironment, which impacts on all myocardial cells [4].

Advanced medical and pharmacological treatments in the setting of HF have made great strides, but heart transplantation and ventricular assist devices are the only conclusive therapies for end-stage patients, albeit strongly limited by organ availability, immunological issues, and ultimately cost. Therefore, research has focused on the development of alternative therapies. Cardiac regenerative medicine, including cardiac cell therapy (CCT) [5,6,7], aims at achieving regeneration for compensating parenchymal loss and recovering heart function. Decades of preclinical and clinical research, though, have highlighted the importance of an integrated perspective on tissue regeneration through CCT [8], where intercellular and microenvironmental crosstalk determines indeed the therapeutic outcome. In the context of CCT, regenerative cells need a supporting microenvironment (for example in terms of ECM) to optimize this crosstalk, to boost regeneration and angiogenesis, while counteracting fibrosis [9,10,11].

Among resident stromal cells, a population of cardiac primitive cells (CPCs) is traceable in the adult human heart by means of different criteria [12,13,14,15,16], which can mediate CCT benefits through multiple biological mechanisms, including direct regeneration and, more importantly, angiogenic, anti-fibrotic, cytoprotective, and anti-inflammatory effects [17,18]. Multiple pathways and signals have been reported to affect the phenotype of CPCs [19,20,21,22,23,24,25]. Biological and functional changes occurring during cardiac adverse remodeling affect both ECM composition and architecture. The consequent disrupted biological and mechano-sensing signaling can, thus, interfere with the balance between cardiogenic and pro-fibrotic potential of resident or transplanted CPCs [9,26,27,28]. Therefore, it is important to understand how cardiac ECM may regulate the cell phenotype of both endogenous and transplanted CPCs, and how this interaction may affect the balance between regeneration and fibrosis in the damaged myocardium. Furthermore, physiologically relevant in vitro settings are needed both to investigate this crosstalk with the microenvironment, and also as bioactive substrates for CPC culture, that are crucial for translational purposes [29,30,31]. In fact, organ regeneration based on decellularized native ECM scaffolds [32,33] holds great promise for effective cardiac tissue engineering approaches, despite being an ambitious goal.

We have previously reported that cardiac primitive cells differentially respond in vitro to ECM synthetized by cardiac fibroblasts isolated from normal or pathological hearts. Specifically, ECM synthesized by HF-derived fibroblasts negatively affects CPC proliferation, migration, and secretion of trophic and anti-remodeling cytokines [34,35], suggesting a detrimental crosstalk between the CPC compartment and ECM synthetized by fibroblasts during HF. Data on more complex microenvironments, though, retaining both physiological ECM composition and architecture, are still limited.

Accordingly, the aim of the present study was to investigate the effects of human decellularized native ECM from normal or pathological HF hearts on a population of human cardiac stromal cells, including CPCs. Our results will be useful to better understand the influence of pathological ECM remodeling on cardiac stromal populations, also for translational purposes on suitable cell product candidates for heart regeneration.

## 2. Results

**Figure 1:**
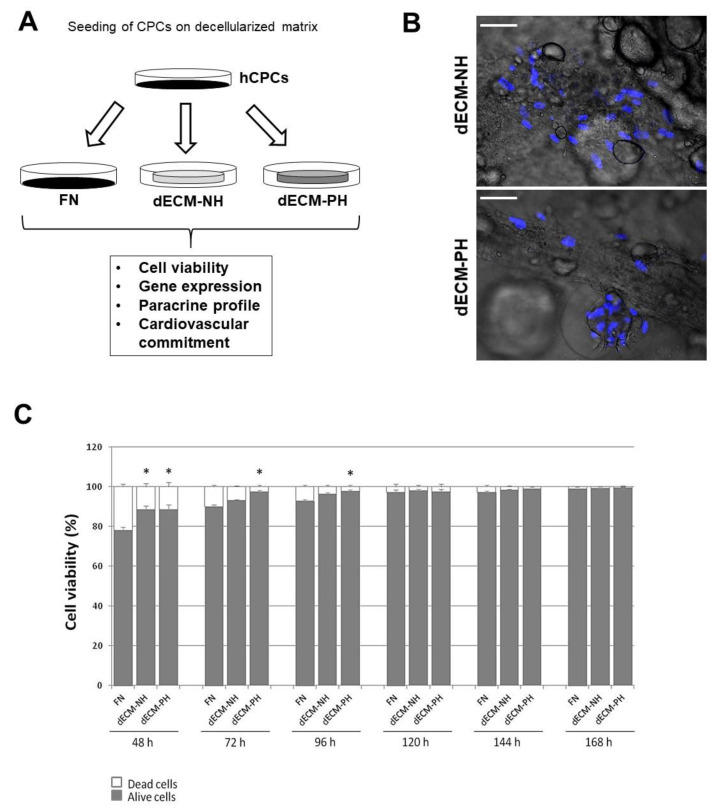
Experimental design, cell plating, and viability. (**A**) Schematic representation of the cell culture and seeding procedures on fibronectin coating (FN) and decellularized extracellular matrix (dECM) from normal hearts (NH) or pathological hearts (PH). (**B**) Representative merge live images (contrast phase + Hoescht fluorescence for nuclei) of human cardiac primitive stromal cells (hCPCs) 24 h after seeding on dECM-NH or dECM-PH. Scale bars = 20 µm. (**C**) Cell viability time-course by Trypan Blue exclusion assay of CPCs seeded on FN, dECM-NH, or dECM-PH up to 1 week. *: *p* < 0.05 versus corresponding FN control.

Decellularized native extracellular matrix sections obtained from normal or pathological hearts with advanced HF (dECM-NH and dECM-PH, respectively) were seeded with human CPCs isolated from IHD patients (Figure 1A,B) and cultured up to 7 days, with standard culture on fibronectin (FN)-coating as control. Cell viability of CPCs cultured on cardiac dECMs or on FN-coated dishes was quantified daily using a trypan blue exclusion assay (Figure 1C). Cell death rate 2 days after seeding was significantly lower on both dECMs compared to FN (21.82 ± 1.36%), without any statistically significant difference between dECM-NH and dECM-PH (11.45 ± 1.68% and 11.41 ± 2.32%, respectively). Between 3 and 4 days after cell seeding, cell death rate resulted significantly lower on dECM-PH than FN (2.42 ± 0.75% versus 10.01 ± 0.85% after 3 days, and 2.16 ± 0.73% versus 7.27 ± 0.82% after 4 days). Nonetheless, starting at 5 days after seeding, cell death rate dramatically decreased on all substrates until it reached values as low as 0.4% after 7 days of culture, without any significant differences among dECMs and FN (1.05 ± 0.03% on FN, 0.88 ± 0.02% on dECM-NH, and 0.41 ± 0.36% on dECM-PH after 168 h). Therefore, cell viability increased with time on all substrates with differences that were statistically significant only until 4 days after cell seeding (Figure 1C).

**Figure 2:**
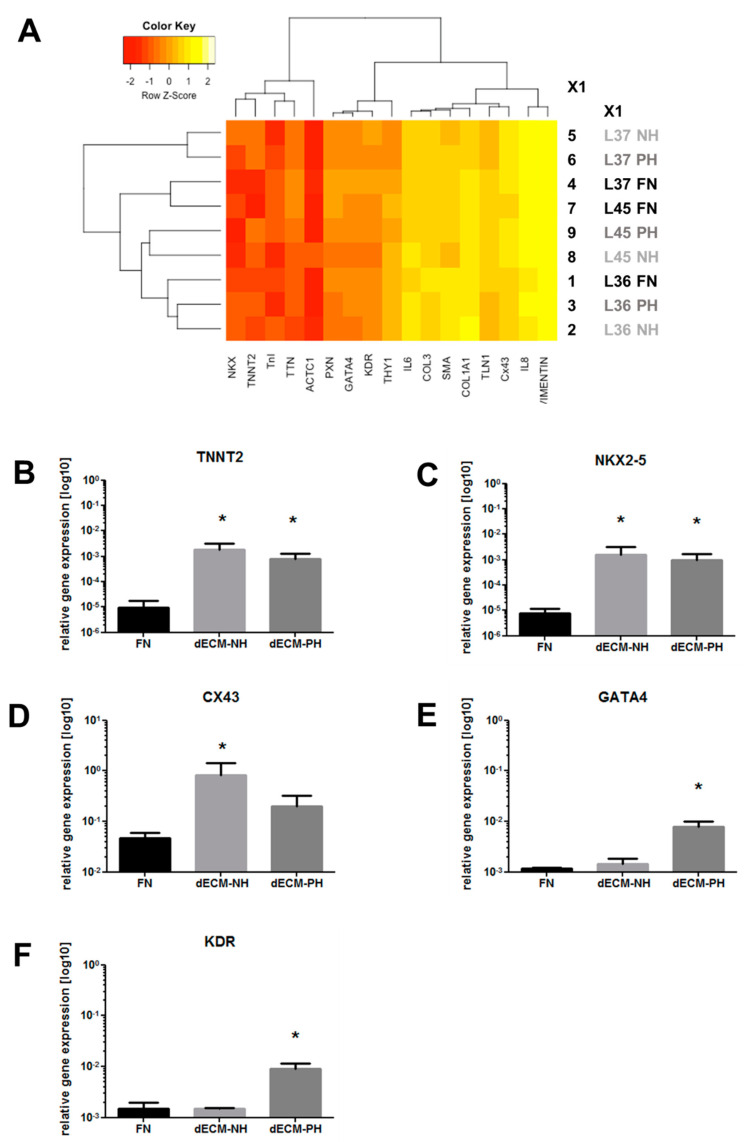
Gene expression analysis of CPCs seeded on different substrates. (**A**) Heatmap and hierarchical clustering analysis of the complete panel of genes analyzed by real-time PCR. Single gene analysis was then performed for the expression levels of significantly modulated genes, that is, troponin T (TNNT2) (**B**), NK2 Homeobox 5 (NKX2-5) (**C**), connexin 43 (CX43) (**D**), GATA4 (**E**), and kinase insert domain receptor (KDR/VEGFR2) (**F**), in CPCs cultured on fibronectin coating (FN), or on decellularized extracellular matrix (dECM) from normal hearts (NH) or pathological hearts (PH). *n* = 3–6. *: *p* < 0.05 vs. FN.

A comprehensive panel of genes was analyzed by realtime PCR for expression levels in CPCs after 7 days of culture on the different substrates. Hierarchical clustering analysis did not evidence any significant difference among sample groups (Figure 2A). Nonetheless, analysis on specific genes evidenced a statistically significant increase in the expression of cardiac-specific transcription factor NK2 Homeobox 5 (NKX2-5) and Troponin T (TNNT2) on both dECM substrates compared with FN control (Figure 2B,C). Upregulation of the gap junction protein connexin 43 (CX43) was significant only on dECM-NH sections (Figure 2D), while GATA4 and kinase insert domain receptor (KDR, also named vascular endothelial growth factor receptor 2, VEGFR2) were significantly upregulated only on dECM-PH sections (Figure 2E,F). Other genes analyzed, but not modulated in these conditions, include adhesion and ECM proteins, such as Vinculin and Collagen I (Supplementary Figure S1).

**Figure 3:**
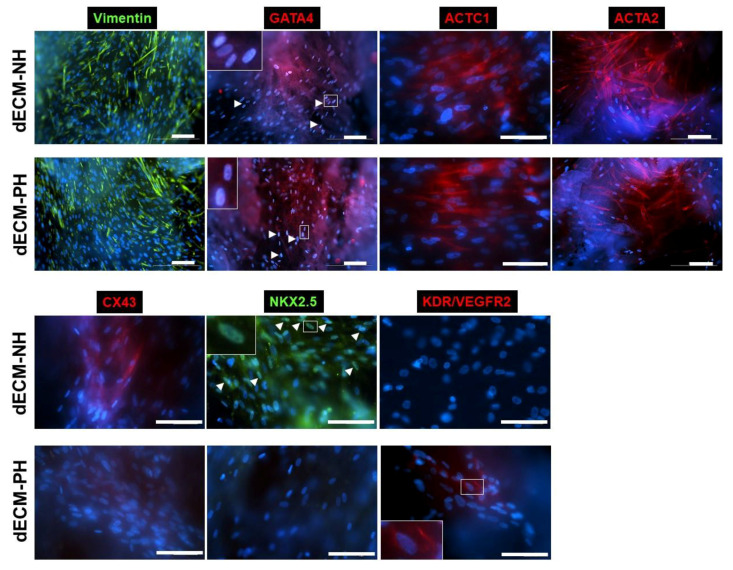
Immunofluorescence analysis of CPCs seeded on dECMs. Representative fluorescence microscope images of stained CPCs cultured for 1 week on normal (NH) or pathological (PH) dECMs. Inserts in panels show higher magnification details. Scale bars = 100 µm. ACTC1: cardiac muscle alpha actin. ACTA2: smooth muscle alpha (α)-2 actin. CX43: connexin 43. NKX2.5: NK2 Homeobox 5. KDR: kinase insert domain receptor. Arrowheads: transcription factor-positive nuclei.

To assess possible differences in the ability of dECMs to direct the phenotype of CPCs, we evaluated the expression of typical markers of cardiomyocytes, endothelial, mesenchymal, and smooth muscle cells by immunofluorescence (Figure 3). Consistently with real-time PCR data, immunofluorescence analysis revealed that CPCs cultured on cardiac dECM, regardless of its derivation, expressed Vimentin, GATA4, Cardiac alpha-Actin (ACTC1), and Smooth Muscle Actin (ACTA2). However, CX43 and NKX2.5 were detectable by immunofluorescence staining only when CPCs were cultured on dECM-NH, while their expression on dECM-PH was not detected in these conditions. This apparent difference from NKX2.5 gene expression data suggests regulation at post-transcriptional level, as previously described [36]. Conversely, CPCs were immunopositive for KDR/VEGFR2 only when cultured on dECM-PH, consistently with gene expression levels (Figure 2F). This latter staining, though, was compatible with both intracellular and membrane signal.

**Figure 4:**
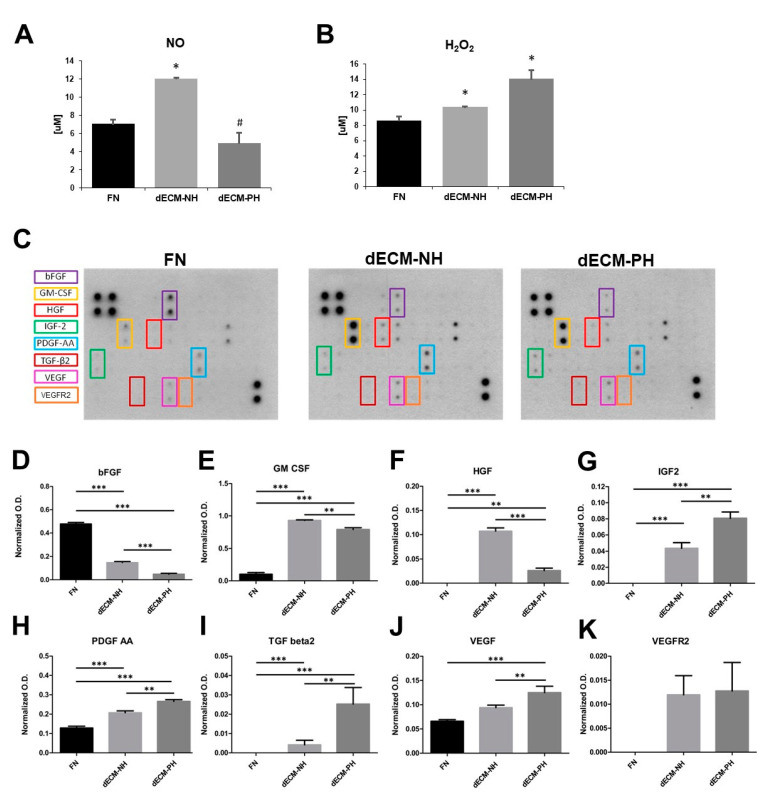
Analysis of cell culture supernatants. Quantification of nitric oxide (NO) (**A**) and hydrogen peroxide (H_2_O_2_) (**B**) in conditioned media from CPCs cultured on fibronectin coating (FN), or on decellularized extracellular matrix (dECM) from normal hearts (NH) or pathological hearts (PH). *n* = 3. *: *p* < 0.05 vs. FN. #: *p* < 0.05 vs. NH. (**C**) Representative protein array membranes blotted with CMs from different culture conditions. (**D**–**K**) Histograms with specific quantification by densitometry of selected cytokines: basic fibroblast growth factor (bFGF), granulocyte-macrophage colony-stimulating factor (GM-CSF), hepatocyte growth factor (HGF), insulin-like growth factor 2 (IGF-2), platelet-derived growth factor AA (PDGF-AA), transforming growth factor beta 2 (TGF-β2), vascular endothelial growth factor (VEGF), and KDR/VEGFR2. *n* = 3. **: *p* < 0.01. ***: *p* < 0.001.

Nitric oxide (NO, a pro-angiogenic mediator), hydrogen peroxide (H_2_O_2_, a reactive oxygen species), and growth factors release were assessed in 48 hour-conditioned media (CM) from all culture conditions. NO levels were significantly higher in CM from dECM-NH cultured cells, compared to both dECM-PH sections and control substrates (Figure 4A), while H_2_O_2_ concentration was increased in CMs collected from both dECMs versus FN, with higher levels on dECM-PH cultures (Figure 4B).

The comparative screening analysis of the profile of cytokines and growth factors released by CPCs on different substrates was performed by protein array (Figure 4C). The analysis revealed that lower amounts of basic fibroblast growth factor (bFGF) were released on both dECMs compared to CPCs cultured on fibronectin, with a significant difference also in dECM-PH versus dECM-NH (Figure 4D). Moreover, CPCs cultured on both normal and pathological cardiac dECM released significantly higher amounts of granulocyte-macrophage colony-stimulating factor (GM-CSF) (Figure 4E) and hepatocyte growth factor (HGF) (Figure 4F), this latter being also significantly upregulated in dECM-NH versus PH. Additionally, the release of insulin-like growth factor 2 (IGF-2) (Figure 4G), platelet-derived growth factor AA (PDGF-AA) (Figure 4H), transforming growth factor beta 2 (TGF-β2) (Figure 4I), and vascular endothelial growth factor (VEGF) (Figure 4J) increased significantly on both dECMs, particularly when CPCs were cultured on dECM-PH. Interestingly, very low levels of the receptor KDR/VEGFR2 were detectable in CMs from dECM cultures by protein array (Figure 4C,K), suggesting the presence of a secreted isoform.

**Figure 5:**
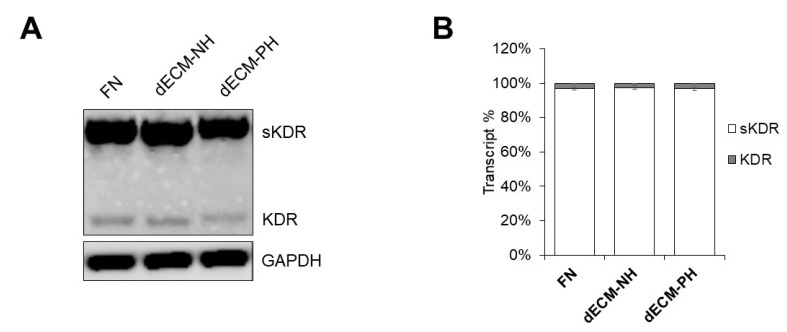
PCR analysis of KDR/VEGFR2 isoforms. Analysis of the relative abundance of mRNA transcripts for the soluble (sKDR) or membrane (KDR) form of KDR/VEGFR2. A representative image is shown (**A**) of an agarose gel run with PCR products evidencing the two KDR/VEGFR2 transcripts, with the housekeeping gene GAPDH as loading control. In order to be able to appreciate the very dim lower band, the gel image is shown as negative. Densitometric quantification (**B**) confirmed no difference among samples in transcript abundance (*n* = 3). sKDR: soluble form of KDR. FN: fibronectin coating. dECM-NH: decellularized extracellular matrix from normal hearts. dECM-PH: decellularized extracellular matrix from pathological hearts.

This last result, associated with the corresponding real-time PCR (Figure 2F) and immunofluorescence data (Figure 3), required further investigation. We performed a PCR with specific primer design to distinguish transcripts for soluble and membrane bound KDR/VEGFR2, in order to understand which isoform was upregulated on pathological dECM. Electrophoretic analysis and densitometric quantification demonstrated that virtually all KDR/VEGFR2 transcripts corresponded to the decoy receptor soluble form (sKDR) in all samples (Figure 5A,B), which is known to sequester bioavailable VEGF, thus reducing its paracrine angiogenic action [37]. We attempted a more accurate quantification of secreted KDR/VEGFR2 by ELISA, but the assay detection limit was not low enough to allow a reliable comparison among groups in our samples. 

**Figure 6:**
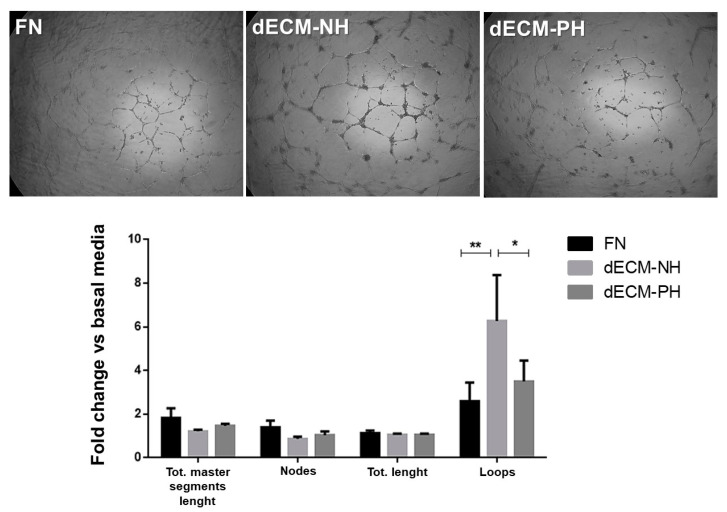
Analysis of pro-angiogenic potential of conditioned media from different substrates. Representative images are shown of human umbilical vein endothelial cells (HUVECs) plated on Matrigel for 18 h, and differentially cultured in conditioned media by CPCs on fibronectin coating (FN), or on decellularized extracellular matrix (dECM) from normal hearts (NH) or pathological hearts (PH). Different features of HUVECs angiogenic capacity have been quantified by image analysis and plotted in the histogram: total master segments length, number of nodes, total tubes length, and number of closed loops. *n* = 8. *: *p* < 0.05. **: *p* < 0.01. Magnification 65×.

To test the pro-angiogenic paracrine function on differential substrates, CPC conditioned media (CM) were used to culture human umbilical vein endothelial cells (HUVECs) on Matrigel overnight. The quantification of total tubes length, total master segments length, and nodes were similar in all conditions, while in CM from dECM-NH cultures the number of closed loops was significantly higher than CM from pathological sections or control conditions (Figure 6). These results were consistent with a higher release of the pro-angiogenic mediator NO on dECM-NH (Figure 4A), and with KDR/VEGFR2 data (Figure 2F, Figure 3 and Figure 5), indicating overall full angiogenic capacity by HUVECs only when exposed to CM from CPCs cultured on dECM-NH.

## 3. Discussion

The ECM is a dynamic and complex environment capable of significantly regulating cell behavior [38,39,40]. Understanding how the interplay between ECM and CPCs can affect the balance between regeneration and fibrosis will provide new insights on myocardial microenvironment, and innovative thrust towards the improvement of regenerative therapies. We have previously shown that the in vitro substrate produced by human cardiac fibroblasts isolated from failing hearts brings CPCs to a pro-fibrotic and pro-remodeling paracrine profile, compared to the substrate synthesized by cardiac fibroblasts derived from healthy hearts [35]. To perform a more comprehensive evaluation of the effects of the naturally occurring cardiac ECM on cardiac stromal cells, in the present study we used three-dimensional decellularized human cardiac ECM.

The survival and phenotype of CPCs was significantly affected by the dECMs in vitro (Figure 1C, Figure 2 and Figure 3). As compared to standard FN culture conditions, cardiac commitment-related genes were either significantly upregulated on both dECMs (NKX2.5 and TNNT2), or exclusively on dECM-NH (CX43) or dECM-PH (GATA4 and KDR/VEGFR2). These results show that dECMs represent a more physiological and cardiogenic culture microenvironment for CPCs compared to standard 2D culture conditions. Nonetheless, protein expression for NKX2.5 and CX43 was detectable by immunofluorescence only on dECM-NH (Figure 3), suggesting a stronger cardiac commitment drive only on the physiologic healthy architecture and composition of dECM-NH.

Interestingly, gene expression of the endothelial-related receptor KDR/VEGFR2 was significantly upregulated in CPCs cultured on pathological dECM sections (Figure 2F). Specific PCR experiments revealed that the proportion of transcript for membrane-associated KDR/VEGFR2 was negligible in all conditions, while its soluble form was the absolute majority in all conditions (Figure 5). Moreover, KDR/VEGFR2 protein was detectable by immunofluorescence only on dECM-PH; albeit, we could not distinguish between intracellular and membrane signals in our conditions. Overall, data suggest that CPCs cultured on pathological dECM express significantly higher levels of decoy KDR/VEGFR2 compared to dECM-NH. Despite consistent VEGF release also on dECM-PH (Figure 4J), functional results (Figure 6) are coherent with sequestering and low bioavailability of VEGF. Moreover, cells cultured on pathological dECM sections also released significantly lower levels of NO (a well-known mediator of angiogenesis [41,42,43]), HGF (an angiogenic growth factor with multiple functions, including inhibiting fibrosis and activating tissue regeneration [44]), and GM-CSF, which also possess pro-angiogenic properties [45]. These changes were consistently associated to reduced pro-angiogenic paracrine support to endothelial cells: in fact, HUVEC angiogenic functional assay demonstrated that only conditioned media from dECM-NH was able to stimulate complete angiogenesis within 18 h, including mature closed loops formation (Figure 6). Overall, our combined observation of decreased NO, increased decoy KDR/VEGFR2 expression, and reduced pro-angiogenic activity points to the existence of a block in VEGF-mediated angiogenic signaling in CPCs exposed to pathological ECM.

It is well established that GATA4 plays an important role in cardiac hypertrophy, acting as a rescue agent. In stress conditions, GATA4 is overexpressed in cardiomyocytes, leading to compensatory paracrine mechanisms [46]. In our conditions, GATA4 gene expression was significantly higher on dECM-PH; albeit, no qualitative difference was appreciable by immunofluorescence staining. Moreover, conditioned media analyses underlined that H_2_O_2_ release in CM from CPCs on pathological dECM was higher than the other two conditions. H_2_O_2_ can act as a rescue agent in stress conditions; in fact, it is released from myocardial cells during ischemia/reperfusion injury, and it is able to interact with membrane KDR/VEGFR2 and stimulate tissue vascularization [47]. For the evidence presented above, we can hypothesize that when embedded in pathological dECM, CPCs are detrimentally affected by the altered microenvironment, and consequently forced to activate a “rescue-like plan”. This plan is nonetheless defective due to the increased production of the soluble form of KDR/VEGFR2, which indeed acts as a decoy for secreted VEGF, paralleled to reduced production of NO, impairing overall pro-angiogenic signals. These results suggest a novel mechanism by which the cardiac stromal primitive compartment may partially lose its paracrine ability to sustain angiogenesis in IHD and HF myocardium subjected to pathological ECM remodeling.

Concerning the paracrine profile, the microenvironment created by dECM-PH seemed also to support pro-fibrotic signaling from CPCs. In particular, PDGF-AA and TGF-β2 release in CM from dECM-PH was increased compared to dECM-NH. PDGF-AA promotes fibrosis through multiple mechanisms, such as inducing proliferation, myofibroblast polarization, and collagen I synthesis in cardiac fibroblasts [48,49]. TGFβ2, instead, is involved in inducing endothelial-to-mesenchymal transition (EndMT), which is a main mechanism of cardiac fibrosis [50,51]. Basic FGF release, instead, was significantly reduced on dECMs, particularly on dECM-PH; this growth factor is known to attenuate myofibroblast-mediated ECM remodeling and fibrosis progression in the heart [52], and its signaling sustains cardiomyocyte homeostasis through CX43 phosphorylation [53]. Finally, high IGF-2 levels have been associated to reduced recovery from HF due to reverse remodeling [54]; interestingly, CPCs cultured on pathological dECM indeed released higher amounts of IGF-2, further supporting a pro-fibrotic paracrine profile from CPCs in this condition. Based on our results, one might speculate that TGFβ2 secreted by CPCs in the presence of dECM-PH may sustain interstitial fibrosis, that is, the typical post-infarction compensatory response of the uninjured myocardium, remote to the reparative fibrosis [55]. Moreover, in a kind of vicious circle, CPCs respond to pathological remodeled cardiac ECM, likely through signals transduced by β1 integrin [56,57], releasing TGFβ2 that, in turn, induces the activation of resident fibroblasts, promotes the persistence of myofibroblasts, and induces the synthesis of fibrillar collagens, thus possibly further worsening ventricular fibrosis and compliance. Despite this shift in paracrine signaling, though, CPCs did not display features of direct contribution to ECM deposition, as for collagen expression levels observed (Supplementary Figure S1).

In conclusion, CPCs exposed in vitro to dECM-PH from HF myocardium displayed partially reduced cardiac commitment drive, defective paracrine support to angiogenesis, and increased release of pro-fibrotic cytokines, despite displaying some features of a rescue-like mechanism, similar to those described in vivo in pathological conditions. These observations shed novel insights on the crosstalk between ECM remodeling and cardiac stromal primitive cells, suggesting also caution and limitations in the use of non-healthy decellularized myocardium for cardiac tissue engineering approaches.

## 4. Materials and Methods

### 4.1. Decellularized Extracellular Matrix (dECM) Sections Production 

Cardiac tissue samples were collected from normal and pathological adult human hearts. Waste fragments of atrial appendages from heart of donors (*n* = 6, mean age 36.4 ± 8.9 years) who died for reasons other than cardiovascular diseases, and samples from atrial appendages of explanted hearts of patients (*n* = 6; mean age 59.3 ± 5.2 years; 4 males, 2 females; mean ejection fraction 16.7 ± 2.6%) with end-stage HF associated with ischemic cardiomyopathy, were collected during heart transplant procedures performed at the Monaldi Hospital (Naples, Italy). Specimens were snap-frozen and stored at −80 °C. Informed consent for use of heart tissue for experimental studies was obtained from all patients, and samples were collected and classified without patient identifiers, in accordance with protocols approved by the Ethical Committee of Monaldi Hospital and the “Federico II” Hospital (79/18, May 11th, 2018), and in conformity with the principles outlined in the Declaration of Helsinki. Frozen specimens were mounted on a cryostat chuck using Tissue Freezing Medium (Leica Microsystems, Wetzlar, Germany). Then, 200 μm thick sections were cut by a Leica CM1950 cryostat (Leica Microsystems, Wetzlar, Germany). Cryosections were collected in sterile 15 mL plastic tubes containing sterile sodium chloride solution (Sigma-Aldrich, St. Louis, MO, USA). Afterwards, cryosections were decellularized, as previously described [58]. Briefly, cryosections were immersed in a 1% SDS, 1% Triton solution in bidistilled water for 24 h, then rinsed for 24 h in antibiotic solution (100 U/mL penicillin, 50 U/mL streptomycin, and 0.25 μg/mL amphotericin B, all from Sigma-Aldrich, St. Louis, MO, USA) in PBS and finally for an additional 30 min in sterile bi-distilled water. All steps were performed with agitation on an orbital shaker. Sections of cardiac dECM were then mounted on sterile 35 mm cell culture dishes and on 96-well cell culture plates, sterilized by exposure to ultraviolet radiation for two cycles of 20 min each, and rehydrated for 5 days with Iscove’s modified Dulbecco’s medium (IMDM) supplemented with 20% FBS (both from Sigma-Aldrich, St. Louis, MO, USA) and antibiotics, in an incubator at 37 °C with 5% CO_2_. Once rehydrated, sections of dECM from normal and pathological hearts (dECM-NH and dECM-PH, respectively) were used as substrates for cell culture.

### 4.2. Cardiosphere-Derived Cell Culture 

CPCs were derived from 4 donor patients (3 males aged 58, 73 and 74; 1 female aged 63) under beta-blocker therapy [59], and undergoing elective cardiac surgery for IHD. Cells were isolated from right atrial appendage biopsies collected during clinically indicated procedures, after informed consent, and under protocol 2154/15 approved by the Ethical Committee of “Umberto I” Hospital, “La Sapienza” University of Rome. Undifferentiated CPCs were selected by spontaneous spheroid growth, as cardiosphere-derived cells, as previously described [60]. CPCs were seeded on sections of native dECM, either normal (dECM-NH) or pathological (dECM-PH), at a density of 2.5 × 10^5^ cells/cm^2^ of section (Figure 1A). Cells were then cultured for 7 days in complete explant medium (CEM) media: Iscove’s modified Dulbecco’s medium (IMDM) supplemented with 20% FBS, 1% penicillin–streptomycin (all from Sigma-Aldrich, St. Louis, MO, USA), 1%L-glutamine (Lonza, Basel, Switzerland), and 0.1 mM 2-mercaptoethanol (Gibco, Thermo Fisher Scientific, Waltham, MA, USA). Cells grown on fibronectin-coated plates at the same density were used as reference.

### 4.3. Assay of CPC Viability

An amount of 1 × 10^4^ CPCs were seeded on dECM-NH or dECM-PH mounted on 96-well plates and cultured for 7 days under standard culture conditions in the same cell culture medium used for hydration. As a control, 1 × 10^4^ cells were seeded on fibronectin-coated wells. Cells were checked daily by an Olympus CKX41 inverted microscope equipped with a Colorview IIIu digital camera (Olympus Corporation, Tokyo, Japan). Beginning at 48 h after seeding, and then every day for one week, cell death rate was assessed using trypan blue exclusion assay adapting a previously described protocol [58,61]. Specifically, every day cells were detached from a subset of wells in the multi-well plates by incubation with 0.25% trypsin-EDTA solution (Sigma-Aldrich, St. Louis, MO, USA) for 10 min. Detached cells were then stained with trypan blue stain (0.4% in PBS) (Lonza, Walkersville, MD, USA) for 2 min at room temperature and counted using a hemocytometer. The percentage of dead cells and of alive cells over total cells for each time point was expressed as the mean percentages.

### 4.4. Immunostaining and Fluorescence Microscopy Analyses 

An amount of 2.5 × 10^5^ CPCs/cm^2^ were seeded on dECM-NH or dECM-PH mounted on 35 mm culture dishes and cultured under standard culture conditions in the same cell culture medium used for hydration. As a control, 2.5 × 10^5^ cells/cm^2^ were seeded on fibronectin-coated 35 mm culture dishes and cultured in the same conditions. Cells were checked daily by an Olympus CKX41 inverted microscope equipped with a Colorview IIIu digital camera (Olympus Corporation, Tokyo, Japan). After five days of culture, cells were immunostained as previously described [34,62]. Briefly, cells were fixed in 4% paraformaldehyde (Merck Millipore, Darmstadt, Germany) washed in PBS and permeabilized by incubation with 0.1% Triton (Sigma-Aldrich, St. Louis, MO, USA) in PBS. After the blocking of non-specific sites with 10% donkey serum, cells were incubated with the primary antibodies anti-α-sarcomeric actin, kinase insert domain receptor (KDR/VEGFR2), smooth muscle actin (SMA), vimentin (all four from Sigma-Aldrich), Connexin-43 (CX43), GATA binding protein 4 (GATA4), NK2 homeobox 5 (NKX2.5) (all four from Abcam, Cambridge, UK), for 1 h at 37 °C in a humified chamber. After washes in PBS, cells were incubated with matching secondary antibodies conjugated with rhodamine (Jackson Immuno-Research Europe, Newmarket, UK) for 1 h at 37 °C. Nuclei were counterstained with DAPI (Sigma-Aldrich, St. Louis, MO, USA) and mounted in the VECTASHIELD Antifade Mounting Medium (Vector Labs, Burlingame, CA, USA). Microscopic analysis was performed with a Nikon Eclipse Ti-E Microscope DS-Qi2 by NIS Elements software (Nikon Instruments, Tokyo, Japan) with a 20× (for Vimentin, GATA4, ACTA2) or 40x objective (for ACTC1, CX43, NKX2.5, KDR).

### 4.5. RNA Extraction and Real-Time PCR

Total RNA was extracted using the miRNeasy Micro Kit (Qiagen, Hilden, Germany) and quantified using a spectrophotometer. cDNA was synthesized using 0.5μg RNA, with the High Capacity cDNA Reverse Transcription Kit (Life Technologies, Thermo Fisher Scientific, Waltham, MA, USA). Real-time qPCR was performed to assess gene expression, using Power SYBR Green PCR Master Mix (Life Technologies, Thermo Fisher Scientific, Waltham, MA, USA) and standard thermocycling conditions according to the manufacturer’s protocol. The relative ratio for each section versus culture on fibronectin was calculated using the comparative Ct method (2^-ΔΔCt) for each patient’s sample. The set of genes were analyzed, and the primer sequences are listed in Table 1. GAPDH was selected as housekeeping gene, according to the Norm Finder software (MOMA, Aarthus, Denmark).

### 4.6. Soluble KDR/VEGFR2 mRNA Analysis 

We performed semi-quantitative PCR in order to detect the presence of the soluble VEGFR2 isoform. We designed a primer pair specific for the VEGFR2 spanning exon 13 in order to detect the inclusion of this exon by agarose gel electrophoresis [63]. We used GAPDH as reference gene. The primer sequences are listed in Table 1. The PCR was performed using Bestaq DNA polymerase (ABM Inc, New York, NY, USA) as follows: 2′ at 95 °C followed by 35 cycles of 30 s at 95 °C, 45 s at 58 °C, and 45 s at 72 °C. The samples were separated on a 1% agarose gel with Gel Red Nucleic Acid gel stain (Biotium, San Francisco, CA, USA) for bands detection, and the image was acquired and analyzed using Typhoon FLA 9500 imager and analysis software (GE Healthcare Life Sciences, Sheffield, UK).

### 4.7. Conditioned Media Collection 

After 5 days of culture on dECM-NH, dECM-PH, or fibronectin (2.5 × 10^5^ CPCs/cm^2^), media was changed for the last 48 h of culture, in the presence of 0.1% of FBS (Sigma-Aldrich, St. Louis, MO, USA), and then collected to be analyzed for the quantification of cytokines, NO, and H_2_O_2_. Media were centrifuged at 2000rcf for 5 min, and then stored at −80 °C until analysis. Basal non-conditioned media was used as blank control for all assays.

### 4.8. Quantification of H_2_O_2_ and NO 

Assays on conditioned media were performed as previously described [64]. Briefly, H_2_O_2_ was evaluated by a Colorimetric Detection Kit (Arbor Assays, Ann Arbor, MI, USA) and expressed as μmol/L. Intra-assay and inter-assay coefficients of variation were 2.1% and 3.7%, respectively. A colorimetric assay kit (Tema Ricerca, Castenaso, Italy) was used to determine the nitric oxide metabolites nitrite and nitrate (NOx) in cell culture supernatants. Intra-assay and inter-assay coefficients of variation were 2.9% and 1.7%, respectively. Assays were performed according to the manufacturers’ instructions.

### 4.9. Growth Factor Array 

Culture medium was assayed by the Human Growth Factor Array C1 (Raybiotech, Norcross, GA, USA) to simultaneously detect 41 targets. The procedure was performed as previously described [65]. Briefly, array membranes were blocked with blocking buffer for 30 min at room temperature, and then 1 mL of culture medium was added to each membrane and incubated at room temperature for 2.5 h. Membranes were then washed 3 times in wash buffer I, twice in wash buffer II, and then incubated with biotin-conjugated antibody overnight at 4 °C. After further washes, membranes were incubated for 2 h at room temperature with horseradish peroxidase (HRP)-conjugated streptavidin and washed one last time to remove unbound reagents. All incubation steps were performed with agitation on orbital shaker. Membranes were then developed with the detection buffer, exposed to film, and processed by autoradiography. Quantitative comparison of the signal densities was performed following the guidelines supplied with the array protocol. Briefly, images of arrays were scanned, and spot signal densities were obtained using ImageJ software (NIH, Bethesda, WA, USA). The background was then subtracted from the densitometry data, and the obtained values were normalized to the positive control signals.

### 4.10. HUVEC Angiogenic Assay 

Human umbilical vein endothelial cells (HUVECs) were cultured for 18 h on Matrigel-coated 96-well plates (Growth Factor Reduced Matrigel Matrix Phenol Red Free, BD, San Jose, CA, USA) at a density of 2 × 10^4^ cells/well in the presence of the CMs from each condition, using basal non-conditioned media as a negative control. Assay quantification, in terms of total master segment length, total length, and number of loops and nodes, was performed with the Angiogenesis Analyzer Plugin of the ImageJ Software (NIH, Bethesda, WA, USA) on randomly captured images with a 4X objective.

### 4.11. Statistical Analysis 

All results are presented as mean value ± standard error of the mean, unless specified. Significance of difference between any two groups was determined by one-way ANOVA test, while multiple comparison correction was applied when necessary. A final value of *p* < 0.05 was considered significant.

## Figures and Tables

**Table 1:** List of primer sequences for real-time qPCR and semi-quantitative PCR analysis.

Target Gene		Primer Sequence
**CX43**	FW	AGGAGTTCAATCACTTGGCG
	RV	GAGTTTGCCTAAGGCGCTC
**GATA4**	FW	GTTTTTTCCCCTTTGATTTTTGATC
	RV	AACGACGGCAACAACGATAAT
**KDR**	FW	AAAGGGTGGAGGTGACTGAG
	RV	CGGTAGAAGCACTTGTAGGC
**KDR exon 12**	FW	TCAACAAAGTCGGGAGAGGA
**KDR exon 14**	RV	GAGTCTTCTACAAGGGTCTCA
**TnI**	FW	GGACAAGGTGGATGAAGAGA
	RV	AGGGTGGGCCGCTTAAACT
**SMA**	FW	ATGAAGATCCTGACTGAGCG
	RV	GCAGTGGCCATCTCATTTTC
**NKX2-5**	FW	GGTGGAGCTGGAGAAGACAGA
	RV	CGCCGCTCCAGTTCATAG
**TNNT2**	FW	GGAGGAGTCCAAACCAAAGCC
	RV	TCAAAGTCCACTCTCTCTCCATC
**TTN**	FW	GCAAGAGTACCAGCACCTGT
	RV	TCACTGTCGGGGATGGGTAT
**COL1A1**	FW	AAGAGGAAGGCCAAGTCGAG
	RV	CACACGTCTCGGTCATGGTA
**COL3A1**	FW	CATGCCCTACTGGTCCTCAG
	RV	ATAGCCTGCGAGTCCTCCTA
**VIM**	FW	ACCCACTCAAAAAGGACACTTC
	RV	GGTCATCGTGATGCTGAGAA
**PXN**	FW	AAAGTTGCGGGGCATAGAC
	RV	GTAGACTCCAAGTCCGCGAC
**TLN1**	FW	AAGGCACTTTGTGGCTTCAC
	RV	ACTGTGTGGGCTCCACTAGC
**VCL**	FW	ACCTTGAACAACTCCGACTAAC
	RV	AACTCTTCATCCTTTTCCTCTGG
**IL-6**	FW	GGTACATCCTCGACGGCATCT
	RV	GTGCCTCTTTGCTGCTTTCAC
**IL-8**	FW	CTTGGCAGCCTTCCTGATTT
	RV	TTCTTTAGCACTCCTTGGCAAAA
**VEGF**	FW	CTACCTCCACCATGCCAAGT
	RV	CCACTTCGTGATGATTCTGC
**AREG**	FW	AAGGAGAAGCTGAGGAACGAA
	RV	TGGCAGTGACTCCAATGTGA
**HGF**	FW	AAGTGAATACTGCAGACCAATGTG
	RV	AAGGGGAACCAGAGGCATT
**IGF-2**	FW	GGACACCCTCCAGTTCGTCT
	RV	CGGAAACAGCACTCCTCAAC
**PDGFA**	FW	TTTGGACACCAGCCTGAGAG
	RV	AGACAGCGGGGACAGCTT
**GAPDH**	FW	ACAGTCAGCCGCATCTTC
	RV	GCCCAATACGACCAAATCC

## Supplementary Materials

The following are available online at https://www.mdpi.com/1422-0067/21/21/7903/s1, Figure S1: Gene expression levels of adhesion and matrix proteins.

## References

[1] Townsend N., Wilson L., Bhatnagar P., Wickramasinghe K., Rayner M., Nichols M. (2016). Cardiovascular disease in Europe: Epidemiological update 2016. Eur. Heart J..

[2] Schirone L., Forte M., Palmerio S., Yee D., Nocella C., Angelini F., Pagano F., Schiavon S., Bordin A., Carrizzo A. (2017). A review of the molecular mechanisms underlying the development and progression of cardiac remodeling. Oxid. Med. Cell. Longev..

[3] Kajstura J., Leri A., Castaldo C., Nadal-Ginard B., Anversa P. (2004). Myocyte growth in the failing heart. Surg. Clin. North Am..

[4] Frangogiannis N.G. (2017). The extracellular matrix in myocardial injury, repair, and remodeling. J. Clin. Investig..

[5] Marban E. (2018). A mechanistic roadmap for the clinical application of cardiac cell therapies. Nat. Biomed. Eng..

[6] Peruzzi M., De Falco E., Abbate A., Biondi-Zoccai G., Chimenti I., Lotrionte M., Benedetto U., Delewi R., Marullo A.G., Frati G. (2015). State of the Art on the evidence base in cardiac regenerative therapy: Overview of 41 systematic reviews. Biomed. Res. Int..

[7] Marotta P., Cianflone E., Aquila I., Vicinanza C., Scalise M., Marino F., Mancuso T., Torella M., Indolfi C., Torella D. (2018). Combining cell and gene therapy to advance cardiac regeneration. Expert Opin. Biol. Ther..

[8] Emmert M.Y. (2017). Cell-based cardiac regeneration. Eur. Heart J..

[9] Mauretti A., Spaans S., Bax N.A.M., Sahlgren C., Bouten C.V.C. (2017). Cardiac progenitor cells and the interplay with their microenvironment. Stem Cells Int..

[10] Nurzynska D., Iruegas M.E., Castaldo C., Muller-Best P., Di Meglio F. (2013). Application of biotechnology in myocardial regeneration-tissue engineering triad: Cells, scaffolds, and signaling molecules. Biomed. Res. Int..

[11] Castaldo C., Chimenti I. (2018). Cardiac progenitor cells: The matrix has you. Stem Cells Transl. Med..

[12] White A.J., Smith R.R., Matsushita S., Chakravarty T., Czer L.S., Burton K., Schwarz E.R., Davis D.R., Wang Q., Reinsmoen N.L. (2013). Intrinsic cardiac origin of human cardiosphere-derived cells. Eur. Heart J..

[13] Gaetani R., Feyen D.A., Doevendans P.A., Gremmels H., Forte E., Fledderus J.O., Ramjankhan F.Z., Messina E., Sussman M.A., Giacomello A. (2014). Different types of cultured human adult cardiac progenitor cells have a high degree of transcriptome similarity. J. Cell. Mol. Med..

[14] Santini M.P., Forte E., Harvey R.P., Kovacic J.C. (2016). Developmental origin and lineage plasticity of endogenous cardiac stem cells. Development.

[15] Castaldo C., Di Meglio F., Nurzynska D., Romano G., Maiello C., Bancone C., Muller P., Bohm M., Cotrufo M., Montagnani S. (2008). CD117-positive cells in adult human heart are localized in the subepicardium, and their activation is associated with laminin-1 and alpha6 integrin expression. Stem Cells.

[16] Di Meglio F., Castaldo C., Nurzynska D., Miraglia R., Romano V., Russolillo V., Giuseppina L., Vosa C., Montagnani S. (2010). Localization and origin of cardiac CD117-positive cells: Identification of a population of epicardially-derived cells in adult human heart. Ital. J. Anat. Embryol..

[17] Fernandez-Aviles F., Sanz-Ruiz R., Climent A.M., Badimon L., Bolli R., Charron D., Fuster V., Janssens S., Kastrup J., Kim H.S. (2017). Global position paper on cardiovascular regenerative medicine. Eur. Heart J..

[18] Pagano F., Picchio V., Angelini F., Iaccarino A., Peruzzi M., Cavarretta E., Biondi-Zoccai G., Sciarretta S., De Falco E., Chimenti I. (2018). The biological mechanisms of action of cardiac progenitor cell therapy. Curr. Cardiol. Rep..

[19] Chimenti I., Forte E., Angelini F., Giacomello A., Messina E. (2012). From ontogenesis to regeneration: Learning how to instruct adult cardiac progenitor cells. Prog. Mol. Biol. Transl. Sci..

[20] Fabrizi C., Angelini F., Chimenti I., Pompili E., Somma F., Gaetani R., Messina E., Fumagalli L., Giacomello A., Frati G. (2011). Thrombin and thrombin-derived peptides promote proliferation of cardiac progenitor cells in the form of cardiospheres without affecting their differentiation potential. J. Biol. Regul. Homeost. Agents.

[21] Pagano F., Angelini F., Siciliano C., Tasciotti J., Mangino G., De Falco E., Carnevale R., Sciarretta S., Frati G., Chimenti I. (2018). Beta2-adrenergic signaling affects the phenotype of human cardiac progenitor cells through EMT modulation. Pharmacol. Res..

[22] Di Meglio F., Castaldo C., Nurzynska D., Romano V., Miraglia R., Bancone C., Langella G., Vosa C., Montagnani S. (2010). Epithelial-mesenchymal transition of epicardial mesothelium is a source of cardiac CD117-positive stem cells in adult human heart. J. Mol. Cell. Cardiol..

[23] Aghila Rani K.G., Kartha C.C. (2010). Effects of epidermal growth factor on proliferation and migration of cardiosphere-derived cells expanded from adult human heart. Growth Factors.

[24] Di Meglio F., Castaldo C., Nurzynska D., Romano V., Miraglia R., Montagnani S. (2010). Epicardial cells are missing from the surface of hearts with ischemic cardiomyopathy: A useful clue about the self-renewal potential of the adult human heart?. Int. J. Cardiol..

[25] Aquila I., Cianflone E., Scalise M., Marino F., Mancuso T., Filardo A., Smith A.J., Cappetta D., De Angelis A., Urbanek K. (2019). c-kit Haploinsufficiency impairs adult cardiac stem cell growth, myogenicity and myocardial regeneration. Cell Death Dis..

[26] Akhyari P., Kamiya H., Haverich A., Karck M., Lichtenberg A. (2008). Myocardial tissue engineering: The extracellular matrix. Eur. J. Cardiothorac. Surg..

[27] Forte E., Chimenti I., Rosa P., Angelini F., Pagano F., Calogero A., Giacomello A., Messina E. (2017). EMT/MET at the crossroad of stemness, regeneration and oncogenesis: The ying-yang equilibrium recapitulated in cell spheroids. Cancers.

[28] Pesce M., Messina E., Chimenti I., Beltrami A.P. (2017). Cardiac mechanoperception: A life-long story from early beats to aging and failure. Stem Cells Dev..

[29] Pampaloni F., Reynaud E.G., Stelzer E.H. (2007). The third dimension bridges the gap between cell culture and live tissue. Nat. Rev. Mol. Cell Biol..

[30] Chimenti I., Massai D., Morbiducci U., Beltrami A.P., Pesce M., Messina E. (2017). Stem cell spheroids and ex vivo niche modeling: Rationalization and scaling-up. J. Cardiovasc. Transl. Res..

[31] Pagliarosi O., Picchio V., Chimenti I., Messina E., Gaetani R. (2020). Building an artificial cardiac microenvironment: A focus on the extracellular matrix. Front. Cell Dev. Biol..

[32] Hinderer S., Layland S.L., Schenke-Layland K. (2016). ECM and ECM-like materials—biomaterials for applications in regenerative medicine and cancer therapy. Adv. Drug Deliv. Rev..

[33] Belviso I., Romano V., Sacco A.M., Ricci G., Massai D., Cammarota M., Catizone A., Schiraldi C., Nurzynska D., Terzini M. (2020). Decellularized human dermal matrix as a biological scaffold for cardiac repair and regeneration. Front. Bioeng. Biotechnol..

[34] Castaldo C., Di Meglio F., Miraglia R., Sacco A.M., Romano V., Bancone C., Della Corte A., Montagnani S., Nurzynska D. (2013). Cardiac fibroblast-derived extracellular matrix (biomatrix) as a model for the studies of cardiac primitive cell biological properties in normal and pathological adult human heart. Biomed. Res. Int..

[35] Pagano F., Angelini F., Castaldo C., Picchio V., Messina E., Sciarretta S., Maiello C., Biondi-Zoccai G., Frati G., Meglio F.D. (2017). Normal versus pathological cardiac fibroblast-derived extracellular matrix differentially modulates cardiosphere-derived cell paracrine properties and commitment. Stem Cells Int..

[36] Nie J., Jiang M., Zhang X., Tang H., Jin H., Huang X., Yuan B., Zhang C., Lai J.C., Nagamine Y. (2015). Post-transcriptional regulation of Nkx2-5 by RHAU in heart development. Cell Rep..

[37] Ebos J.M., Bocci G., Man S., Thorpe P.E., Hicklin D.J., Zhou D., Jia X., Kerbel R.S. (2004). A naturally occurring soluble form of vascular endothelial growth factor receptor 2 detected in mouse and human plasma. Mol. Cancer Res..

[38] Mishra P.K., Givvimani S., Chavali V., Tyagi S.C. (2013). Cardiac matrix: a clue for future therapy. Biochim. Biophys. Acta.

[39] Gaetani R., Yin C., Srikumar N., Braden R., Doevendans P.A., Sluijter J.P., Christman K.L. (2016). Cardiac-derived extracellular matrix enhances cardiogenic properties of human cardiac progenitor cells. Cell Transpl..

[40] Hastings J.F., Skhinas J.N., Fey D., Croucher D.R., Cox T.R. (2019). The extracellular matrix as a key regulator of intracellular signalling networks. Br. J. Pharmacol..

[41] Cooke J.P., Losordo D.W. (2002). Nitric oxide and angiogenesis. Circulation.

[42] Kroll J., Waltenberger J. (1999). A novel function of VEGF receptor-2 (KDR): Rapid release of nitric oxide in response to VEGF-A stimulation in endothelial cells. Biochem. Biophys. Res. Commun..

[43] Daher Z., Boulay P.L., Desjardins F., Gratton J.P., Claing A. (2010). Vascular endothelial growth factor receptor-2 activates ADP-ribosylation factor 1 to promote endothelial nitric-oxide synthase activation and nitric oxide release from endothelial cells. J. Biol. Chem..

[44] Wang L.S., Wang H., Zhang Q.L., Yang Z.J., Kong F.X., Wu C.T. (2018). Hepatocyte growth factor gene therapy for ischemic diseases. Hum. Gene Ther..

[45] Zhao J., Chen L., Shu B., Tang J., Zhang L., Xie J., Qi S., Xu Y. (2014). Granulocyte/macrophage colony-stimulating factor influences angiogenesis by regulating the coordinated expression of VEGF and the Ang/Tie system. PLoS ONE.

[46] Oka T., Akazawa H., Naito A.T., Komuro I. (2014). Angiogenesis and cardiac hypertrophy: Maintenance of cardiac function and causative roles in heart failure. Circ. Res..

[47] Roy S., Khanna S., Sen C.K. (2008). Redox regulation of the VEGF signaling path and tissue vascularization: Hydrogen peroxide, the common link between physical exercise and cutaneous wound healing. Free Radic. Biol. Med..

[48] Zhao T., Zhao W., Chen Y., Li V.S., Meng W., Sun Y. (2013). Platelet-derived growth factor-D promotes fibrogenesis of cardiac fibroblasts. Am. J. Physiol. Heart Circ. Physiol..

[49] Wang L., Yue Y., Yang X., Fan T., Mei B., Hou J., Liang M., Chen G., Wu Z. (2017). Platelet derived growth factor alpha (PDGFRalpha) induces the activation of cardiac fibroblasts by activating c-kit. Med. Sci. Monit..

[50] Sabbineni H., Verma A., Somanath P.R. (2018). Isoform-specific effects of transforming growth factor beta on endothelial-to-mesenchymal transition. J. Cell. Physiol..

[51] von Gise A., Pu W.T. (2012). Endocardial and epicardial epithelial to mesenchymal transitions in heart development and disease. Circ. Res..

[52] Svystonyuk D.A., Ngu J.M., Mewhort H.E., Lipon B.D., Teng G., Guzzardi D.G., Malik G., Belke D.D., Fedak P.W. (2015). Fibroblast growth factor-2 regulates human cardiac myofibroblast-mediated extracellular matrix remodeling. J. Transl. Med..

[53] Sakurai T., Tsuchida M., Lampe P.D., Murakami M. (2013). Cardiomyocyte FGF signaling is required for Cx43 phosphorylation and cardiac gap junction maintenance. Exp. Cell Res..

[54] Felkin L.E., Lara-Pezzi E.A., Hall J.L., Birks E.J., Barton P.J. (2011). Reverse remodelling and recovery from heart failure are associated with complex patterns of gene expression. J. Cardiovasc. Transl. Res..

[55] Frangogiannis N.G. (2019). Cardiac fibrosis: Cell biological mechanisms, molecular pathways and therapeutic opportunities. Mol. Asp. Med..

[56] Chen C., Li R., Ross R.S., Manso A.M. (2016). Integrins and integrin-related proteins in cardiac fibrosis. J. Mol. Cell. Cardiol..

[57] Xie Y., Ibrahim A., Cheng K., Wu Z., Liang W., Malliaras K., Sun B., Liu W., Shen D., Cheol Cho H. (2014). Importance of cell-cell contact in the therapeutic benefits of cardiosphere-derived cells. Stem Cells.

[58] Di Meglio F., Nurzynska D., Romano V., Miraglia R., Belviso I., Sacco A.M., Barbato V., Di Gennaro M., Granato G., Maiello C. (2017). Optimization of human myocardium decellularization method for the construction of implantable patches. Tissue Eng. Part C Methods.

[59] Chimenti I., Pagano F., Cavarretta E., Angelini F., Peruzzi M., Barretta A., Greco E., De Falco E., Marullo A.G., Sciarretta S. (2016). Beta-blockers treatment of cardiac surgery patients enhances isolation and improves phenotype of cardiosphere-derived cells. Sci. Rep..

[60] Chimenti I., Gaetani R., Forte E., Angelini F., De Falco E., Zoccai G.B., Messina E., Frati G., Giacomello A. (2014). Serum and supplement optimization for EU GMP-compliance in cardiospheres cell culture. J. Cell. Mol. Med..

[61] Belviso I., Sacco A.M., Romano V., Schonauer F., Nurzynska D., Montagnani S., Di Meglio F., Castaldo C. (2020). Isolation of adult human dermal fibroblasts from abdominal skin and generation of induced pluripotent stem cells using a non-integrating method. J. Vis. Exp..

[62] Nurzynska D., Di Meglio F., Romano V., Miraglia R., Sacco A.M., Latino F., Bancone C., Della Corte A., Maiello C., Amarelli C. (2013). Cardiac primitive cells become committed to a cardiac fate in adult human heart with chronic ischemic disease but fail to acquire mature phenotype: Genetic and phenotypic study. Basic Res. Cardiol..

[63] Stevens M., Oltean S. (2019). Modulation of receptor tyrosine kinase activity through alternative splicing of ligands and receptors in the VEGF-A/VEGFR axis. Cells.

[64] Pagano F., Nocella C., Sciarretta S., Fianchini L., Siciliano C., Mangino G., Ibrahim M., De Falco E., Carnevale R., Chimenti I. (2017). Cytoprotective and antioxidant effects of steen solution on human lung spheroids and human endothelial cells. Am. J. Transpl..

[65] Sacco A.M., Belviso I., Romano V., Carfora A., Schonauer F., Nurzynska D., Montagnani S., Di Meglio F., Castaldo C. (2019). Diversity of dermal fibroblasts as major determinant of variability in cell reprogramming. J. Cell. Mol. Med..

