# Supplementary material for: The Microenvironment of Decellularized Extracellular Matrix from Heart Failure Myocardium Alters the Balance between Angiogenic and Fibrotic Signals from Stromal Primitive Cells
Source: Int J Mol Sci. 2020 Oct 24;21(21):7903. doi: 10.3390/ijms21217903 (PMC7662394; doi:10.3390/ijms21217903)
Supplement: Supplementary file 1 [file ijms-21-07903-s001.pdf]

Supplementary material.

The microenvironment of decellularized extracellular matrix from heart failure myocardium alters the balance between angiogenic and fibrotic signals from stromal primitive cells.

Immacolata Belviso [1§], Francesco Angelini [2§], Franca Di Meglio [1], Vittorio Picchio [3], Anna Maria Sacco [1], Cristina Nocella [4], Veronica Romano [1], Daria Nurzynska [1], Giacomo Frati [3,5], Ciro Maiello [6], Elisa Messina [7], Stefania Montagnani [1], Francesca Pagano [8], Clotilde Castaldo [1#], Isotta Chimenti [3,9#] *

[1] Department of Public Health, School of Medicine and Surgery, University of Naples Federico II, Naples, Italy.
[2] Experimental and Clinical Pharmacology Unit, CRO-National Cancer Institute, Aviano (PN), Italy.
[3] Department of Medical Surgical Sciences and Biotechnologies, Sapienza University, Latina, Italy.
[4] Department of Clinical, Internal Medicine, Anesthesiology and Cardiovascular Sciences, Sapienza University, Rome, Italy.
[5] Department of AngioCardioNeurology, IRCCS Neuromed, Pozzilli, Italy.
[6] Department of Cardiovascular Surgery and Transplant, Monaldi Hospital, Naples, Italy.
[7] Department of Maternal Infantile and Urological Sciences, "Umberto I" Hospital, Rome, Italy.
[8] Institute of Biochemistry and Cell Biology, National Council of Research (IBBC-CNR), Monterotondo (RM), Italy.
[9] Mediterranea Cardiocentro, Napoli, Italy.
[§] Equal contribution. [#] Equal contribution.

* Correspondence to: isotta.chimenti@uniroma1.it; tel: +3907731757234. Address: Corso della Repubblica 79, 04100 Latina, Italy.

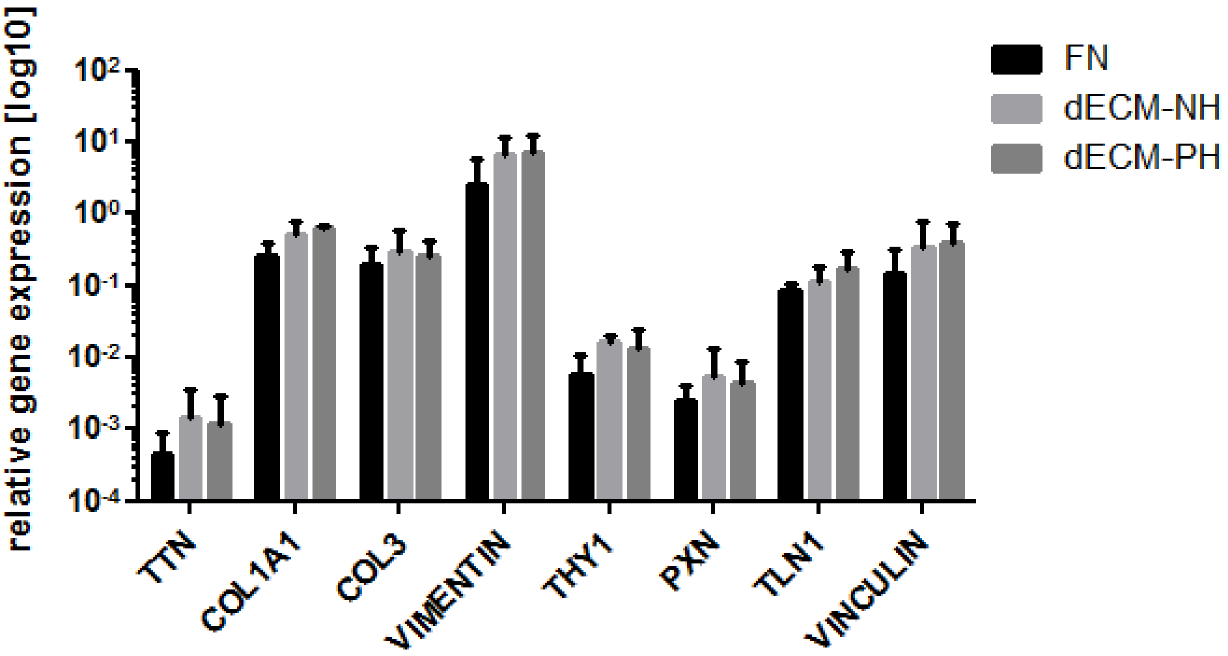

Supplementary figure S1. Gene expression levels of adhesion and matrix proteins. Expression levels of a selection of analyzed genes by realtime PCR. FN: fibronectin coating. dECM-NH: decellularized extracellular matrix from normal hearts. dECM-PH: decellularized extracellular matrix from pathological hearts. TTN: titin. COL1A1: collagen type I. COL3: collagen type III. Thy1: thymus cell antigen 1. PXN: paxillin. TLN1: talin 1.
